# MicroRNA-4516 suppresses pancreatic cancer development via negatively regulating orthodenticle homeobox 1

**DOI:** 10.7150/ijbs.45933

**Published:** 2020-05-18

**Authors:** Shuo Chen, Meng Xu, Jing Zhao, Jiaqi Shen, Junhui Li, Yang Liu, Gang Cao, Jiancang Ma, Weizhou He, Xi Chen, Tao Shan

**Affiliations:** 1 Department of General Surgery, The Second Affiliated Hospital of Xi'an Jiaotong University, Xi'an Jiaotong University, PR China; 2 School of Science, Xi'an Jiaotong University, PR China; 3 School of Life Science, Xiamen University, PR China; 4 Department of Hepatobiliary Surgery, The First Affiliated Hospital of Xi'an Jiaotong University, Xi'an Jiaotong University, PR China

**Keywords:** miR-4516, pancreatic cancer, OTX1, miRNA-based therapy

## Abstract

Pancreatic cancer remains one of the most lethal human cancers without efficient therapeutic strategy. MicoRNAs (miRNAs) are a group of small non-coding RNAs involved in multiple biological processes including tumor development and progression. In this study, we investigated the expression and function of miR-4516 in pancreatic cancer. MiR-4516 was low-expressed in pancreatic cancer tissues and cell lines. Overexpression of miR-4516 inhibited pancreatic cancer cell proliferation, migration and invasion, while promoted cell apoptosis *in vitro*. Further, overexpression of miR-4516 suppressed xenograft pancreatic tumor growth *in vivo*. Bioinformatics analysis was performed and miR-4516 was predicted to negatively regulate orthodenticle homeobox 1 (OTX1) expression by binding to its 3'-UTR. Consistently, OTX1 was highly expressed in pancreatic cancer tissues and cell lines. Knockdown of OTX1 expression suppressed pancreatic cancer cell migration and invasion, with down-regulated MMP2 and MMP9 expression. Moreover, we demonstrated that miR-4516 regulated pancreatic cancer cell growth, migration, invasion and apoptosis via targeting OTX1. Overexpression of OTX1 could partially abrogate the inhibitory effect of miR-4516. Taken together, we conclude that miR-4516 could function as a tumor suppressor via targeting OTX1. These findings suggest that miR-4516/OTX1 axis might be a novel therapeutic target for miRNA-based therapy for pancreatic cancer patients.

## Introduction

Pancreatic cancer is one of the most lethal cancers and ranks 7^th^ leading cause of global cancer deaths in developed countries 1. Though great advances have been achieved in surgical technique, chemotherapy and radiotherapy, the therapeutic outcomes remain poor due to the late diagnosis 2-4. The 5-year relative survivals of pancreatic cancer were only 7.2% in China and the lowest level in all cancers 5. Also, the definite cure strategy is still not available to treat the pancreatic patients with advanced-stages 6, 7. Thus, it is critical to identify the novel diagnostic markers to screen for the patients at early stage and develop new efficient therapeutic strategies to treat late stage pancreatic cancer patients.

MicroRNAs (miRNAs) are a subfamily of non-coding RNAs which post-transcriptionally regulate gene expression and function in various biological process including cancer development 8, 9. MiRNAs play an important role in tumor development and progression, acting as tumor suppressor or oncogene 10. Mounting evidence has demonstrated that multiple miRNAs are involved in pancreatic cancer tumorigenesis and metastasis. Taurine-upregulated gene 1 (TUG1) was reported as miR-299-3p sponge and inhibition of TUG1/miR-299-3p expression dampened pancreatic cancer progression via suppressing the Notch1 signaling pathway 11. Hypoxia-induced miR-646 impaired the stability of Migration and invasion inhibitory protein (MIIP) mRNA and inhibition of miR-646 repressed proliferation and invasion capability of pancreatic cancer cells 12. In contrast, miR-399-5p inhibited the migration and invasion of pancreatic cancer cells via targeting ZNF689 13. Jiang W. et al. reported that miR-101 suppressed epithelial-to-mesenchymal transition via targeting HMGA2 in pancreatic cancer cells. MiR-4516 is a newly identified miRNA involved in autophagy associated with exposure to fine particulate matter 14. A recent study demonstrated that miR-4516 might function as an oncogene in human glioblastoma via targeting PTPN14 15. However, the expression and function of miR-4516 in pancreatic cancer remains largely unknown.

Orthodenticle homeobox 1 (OTX1) is a transcription factor regulating brain and sensory organ development 16, 17. OTX1 was demonstrated to be highly expressed in colorectal cancer and breast cancer as an oncogene 18, 19. Downregulation of OTX1 inhibited cell proliferation, migration and invasion in gastric cancer 20. The role of OTX1 in pancreatic cancer is not clear.

In this study, we demonstrated that miR-4516 was downregulated in pancreatic cancer and cell lines while OTX1 was highly expressed in pancreatic cancer and cell lines. MiR-4516 negatively regulated OTX1 expression by binding to its 3'-UTR. Overexpression of miR-4516 suppressed pancreatic cancer cell proliferation, migration and invasion, while promoted cell apoptosis via regulating OTX1. Together, we demonstrate that miR-4516/OTX1 axis might be a novel therapeutic target for pancreatic cancer treatment.

## Materials and Methods

### Patient Specimens

Paired pancreatic cancer tissues and adjacent noncancerous tissues were obtained from pancreatic patients undergoing surgery in The Second Affiliated Hospital of Xi'an Jiaotong University. Samples were snap-frozen and stored at -80°C until further use. Informed consent was signed by all patients. The study was approved by the Research Ethics Committee of The Second Affiliated Hospital of Xi'an Jiaotong University.

### Cell culture

Human pancreatic cancer cell line SW1990, CAPAN-1, PANC-1 and AsPC-1, and control cell line HPDE6-C7 were obtained from American Type Culture Collection (ATCC, Manassas, VA, USA). All the cell lines were cultured with RPMI1640 medium containing 10% fetal bovine serum (Gibco), 100 U/ml penicillin, 100 μg/ml streptomycin in a cell incubator with 5% CO_2_ at 37°C.

### Transfection

Cell transfection was performed using lipofectamine 3000 (Invitrogen) following the manufacturer's protocol. The miR-NC, miR-4516 mimics, miR-4516 inhibitor and inhibitor negative control were purchased from Ribobio (Guangzhou, China). OTX1 overexpression vector was constructed by cloning the OTX1 ORF into pcDNA3.1 vector.

### RT-qPCR

Total RNA was purified from tissues or cell lines using Trizol (Invitrogen) and reverse-transcribed into complementary DNA using a reverse-transcription kit (Applied Biosystems). The expression of miR-4516 and potential target genes were measured by quantitative PCR (qPCR) using SYBR Green PCR Kit (Applied Biosystems) on an Applied Biosystems Real-time PCR machine. The relative expression was normalized to internal control human U6 RNA or GAPDH RNA using 2^-ΔΔCT^ methods. The primers used in the study were listed below: for miR-4516, 5'-ATGGGAGAAGGGTCGGGG-3'; for OTX1, 5' -CTGCTCTTCCTCAATCAATGG-3' (forward) and 5' -ACCCTGACTTGTCTGTTTCC-3' (reverse); for GAPDH, 5'-CCATCACCATCTTCCAGGAG T-3' (forward) and 5'-GGATGATGTTCTGGAGAGCG-3' (reverse).

### Fluorescence *In Situ* Hybridization

The expression of miR-4516 in pancreatic tissues and control noncancerous tissues were examined by Fluorescence In Situ Hybridization as described before^21^. Paraffin-embedded 4%-PFA-fixed tissues were cut into 6 µm sections and deparaffinized. The antigen was retrieved by boiling in citric acid buffer in a water bath for 20 min. Proteinase K (200 μL; Servicebio, Wuhan, China) in PBS was added to the sections in a humidified chamber, which were then incubated for 25 min at 37 °C and washed twice with PBS for 5 min each. Prehybridization buffer (100 µL; Servicebio) was added to each tissue section. The sections were placed in a hybridization chamber, incubated for 1 h at 37 °C. The prehybridization buffer was replaced with hybridization buffer containing the FAM-labeled miR-4516 probe. The samples were allowed to hybridize overnight at 37 °C. DAPI (Servicebio) was used for nuclear staining.

### CCK-8 assay

Cells were seeded into 96-well plates (2000 cells/well) and incubated for indicated time after transfection. A Cell counting Kit-8 (CCK-8, dojindo) kit was used to evaluate the cell viability at indicated time points. The assay was repeated independently for 3 times.

### EdU staining assay

5-ethynyl-20-deoxyuridine (EdU) assay was performed to assess the DNAY synthesis rate using EdU assay Kit (Ribobio, Guangzhou, China). The staining was recorded and analyzed with a microscopy. The ratio of EdU positive cells (Green) to DAPI-stained cells (Blue) was analyzed to calculate the cell proliferation rate.

### Cell apoptosis assay

Cells were collected 72 hours post transfection and stained with FITC-Annexin V/Propidium Iodide (Sigma). Cell apoptosis was analyzed using flow cytometry and Annexin V+PI- cells were defined as apoptotic cells.

### Transwell assay

Cell invasion and migration was evaluated using Transwell chambers with or without precoated Matrigel layer. Briefly, 30000 cells were seeded into the upper chamber in serum-free medium, the lower chamber was added with medium containing 30% FBS. After incubation for 48 hours, the migrated or invaded cells were fixed with 4% paraformaldehyde and stained with 0.1% crystal violet. The migrated and invaded cells were counted under a light microscopy.

### Luciferase reporter assay

PANC-1 or AsPC-1 cells were seeded into 96-well plates (4000 cells/well) and transfected with luciferase reporter vector containing WT or mutated 3'-UTR of OTX1 (Constructed by Ribobio Company), together with miR-4516 or miR-NC. After 48 hours, the relative luciferase activity was analyzed using the Dual-luciferase reporter assay system (Promega) following the manual.

### Western Blot

Total protein was prepared from tissues or cultured cells using RIPA buffer (Beyotime, China). Equal amount of protein was separated by SDS-PAGE and transferred to polyvinylidene difluoride memebranes (Millipore). The membrane was incubated with primary antibodies against OTX1 (Abcam, ab25985), β-actin (Abcam, ab8227) and then incubated with HPR conjugated secondary antibody. Protein bands were visualized using chemiluminescence reagents (ECL, Pierce).

### Immunohistochemical staining

The immunohistochemical staining of paraffin-embedded tissues was conducted as previously described^22^. Tissue samples was stained with primary antibody anti-OTX1 (Abcam, ab25985) and further incubated with biotinylated goat anti-rabbit secondary antibody (Cell signaling, #14708). Then 3, 3-diaminobenzidine was used to visualize the OTX1 expression.

### Xenograft tumor model

BALB/C nude mice (5-week old) were obtained from Shanghai SLAC Animal Center (Shanghai, China). PANC-1 cells stably overexpression of miR-4516 or control PANC-1 cells (6 × 10^6^) were inoculated subcutaneously into the right flanks of mice. Tumor growth was monitored every 4 days and tumor volume was calculated as volume (mm3) = length × width^2^/2. Mice were euthanized at day 17 and the tumor weights were assessed. The animal experiments were approved by the Animal Care and Use Committee of the Second Affiliated Hospital of Xi'an Jiaotong University.

### Statistics analysis

All data were analyzed using GraphPad Prism V6 and expressed as mean ± SD. Differences between groups was analyzed using the student *t* test (two groups) or One-way ANOVA (multiple groups) where necessary. A P value < 0.05 was considered statistically significant.

## Results

### MiR-4516 is low-expressed in pancreatic cancer tissues and cell lines

**Figure 1:**
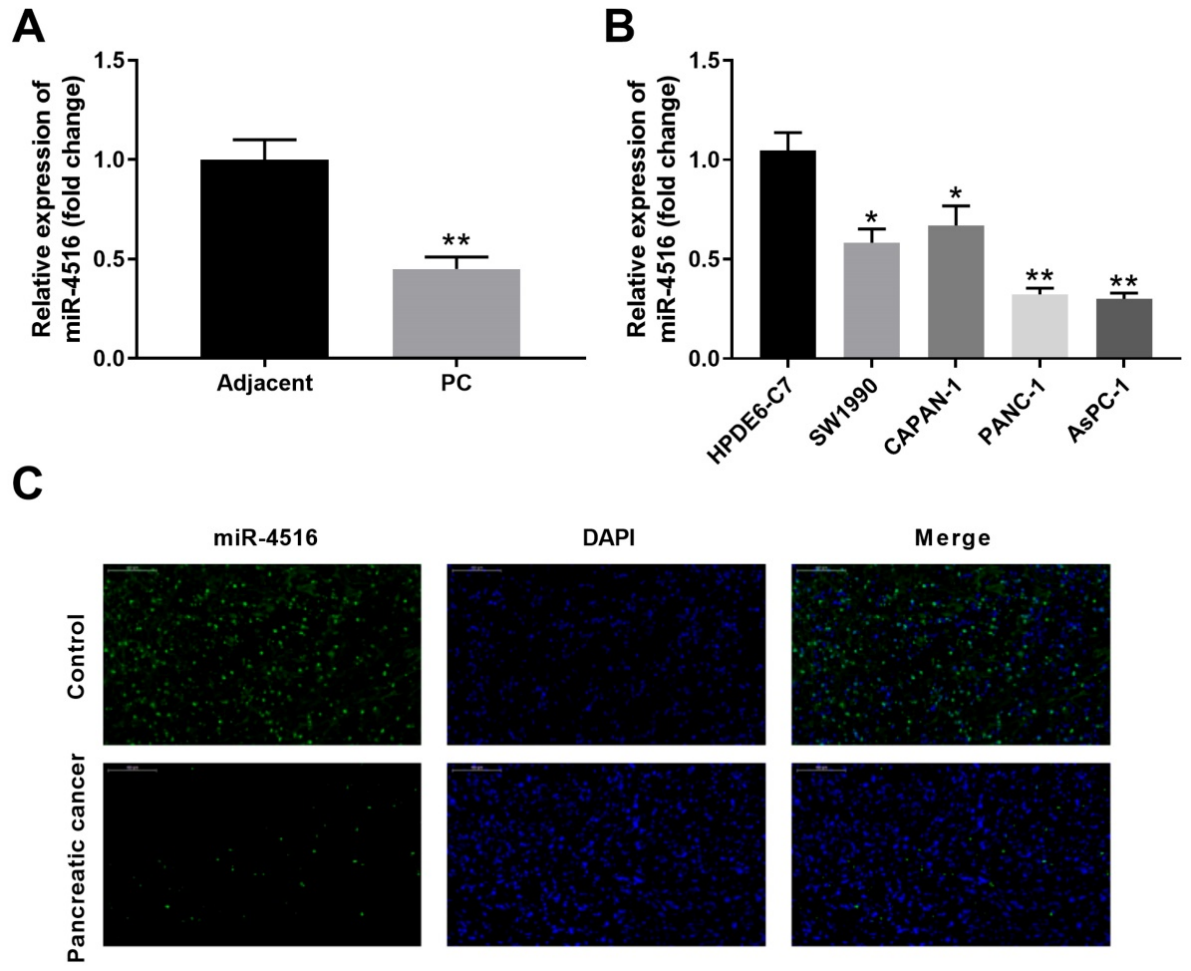
** MiR-4516 is low-expressed in pancreatic cancer tissues and cell lines.** (A) The expression of miR-4516 in 10 paired pancreatic cancer tissues and adjacent noncancerous tissues was examined by qPCR. (B) The expression of miR-4516 in pancreatic cancer cell lines (SW1990, CAPAN-1, PANC-1 and AsPC-1) and control cell line HPDE6-C7 was examined by qPCR. (C) The expression of miR-4516 in paired pancreatic cancer tissues and adjacent noncancerous tissues was examined by in situ hybridization. *, *P* < 0.05; **, *P* < 0.01.

To investigate the expression profile of miR-4516 in pancreatic cancer, we first examined the expression of miR-4516 in 10 paired pancreatic cancer tissues and adjacent noncancerous tissues by qPCR. MiR-4516 is significantly low-expressed in pancreatic cancer tissues compared with that in adjacent noncancerous tissues (**Figure 1A**). In addition, the expression of miR-4516 was much markedly down-regulated in four pancreatic cancer cell lines (SW1990, CAPAN-1, PANC-1 and AsPC-1) in comparison with that in HPDE6-C7 control cell line (**Figure 1B**). We further confirmed the decreased expression of miR-4516 in pancreatic cancer tissues by in situ hybridization (**Figure 1C**). Pancreatic cancer cell lines PANC-1 and AsPC-1, with the lowest expression of miR-4516, were used for the subsequent experiments.

### Overexpression of miR-4516 inhibits pancreatic cell growth, migration and invasion and promotes cell apoptosis in pancreatic cells

**Figure 2:**
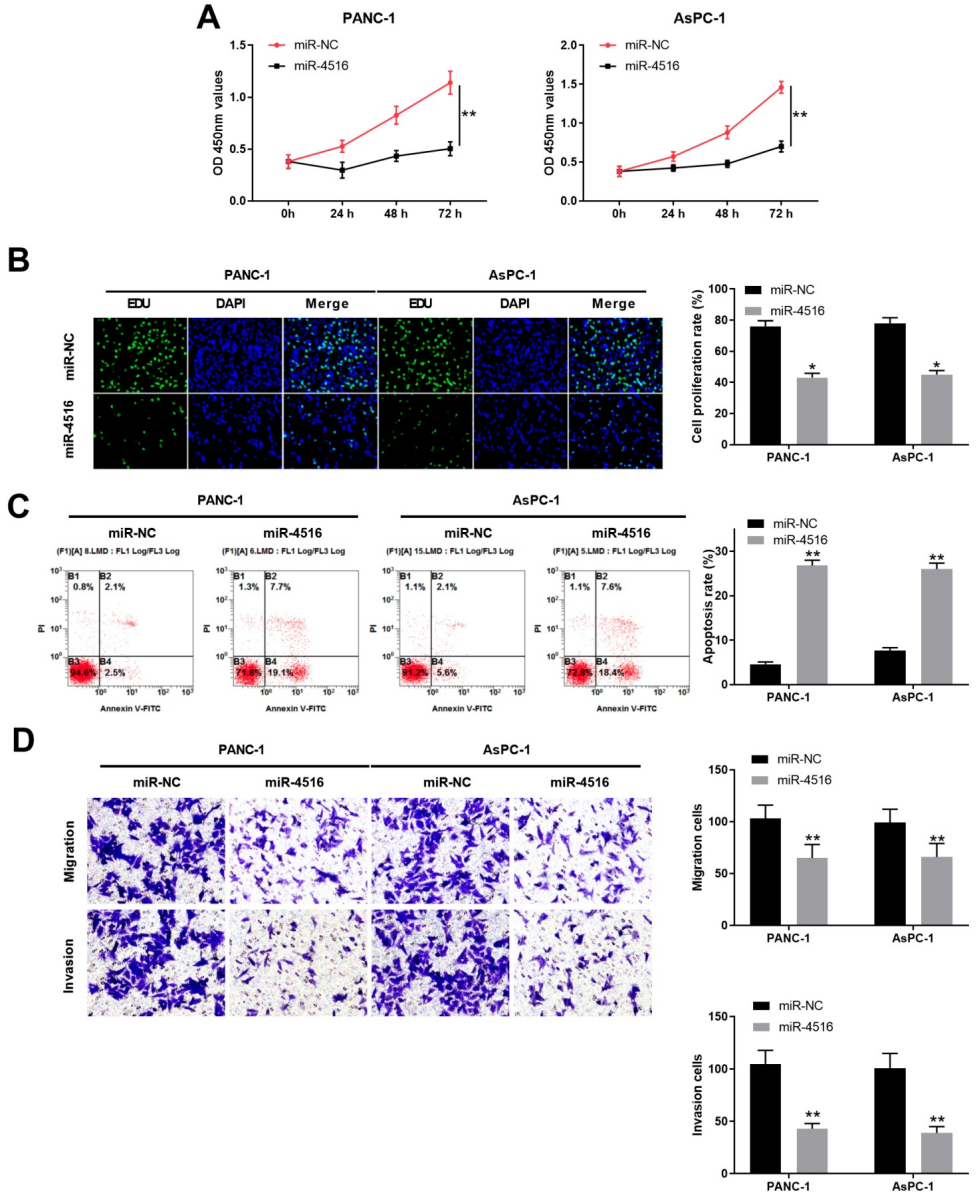
** Overexpression of miR-4516 inhibits pancreatic cell growth, migration and invasion and promotes cell apoptosis in pancreatic cells.** PANC-1 or AsPC-1 cells were transfected with miR-4516 mimics or negative control miR-NC. (A) Cell viability was analyzed by CCK-8 assay at indicated time points. (B) DNA synthesis was analyzed by EdU/DAPI double staining. (C) Cell apoptosis was analyzed by Annexin V/PI double staining. (D) Cell migration and invasion was analyzed by transwell assay. *, *P* < 0.05; **, *P* < 0.01.

To explore the function of miR-4516 in pancreatic cancer, we transfected PANC-1 and AsPC-1 cells with miR-4516 mimics to overexpression miR-4516. As shown in **Figure 2A-2B**, CCK-8 cell proliferation assay and EdU staining assay demonstrated that overexpression of miR-4516 significantly repressed cell growth and DNA synthesis. In contrast, cell apoptosis was remarkably enhanced in PANC-1 and AsPC-1 cells transfected with miR-4516 mimics (**Figure 2C**). Furthermore, cell migration and invasion capability of PANC-1 and AsPC-1 cells transfected with miR-4516 mimics were notably weakened compared with that in cells transfected with negative control miR-NC (**Figure 2D**).

### Overexpression of miR-4516 inhibits pancreatic xenograft tumor growth *in vivo*

**Figure 3:**
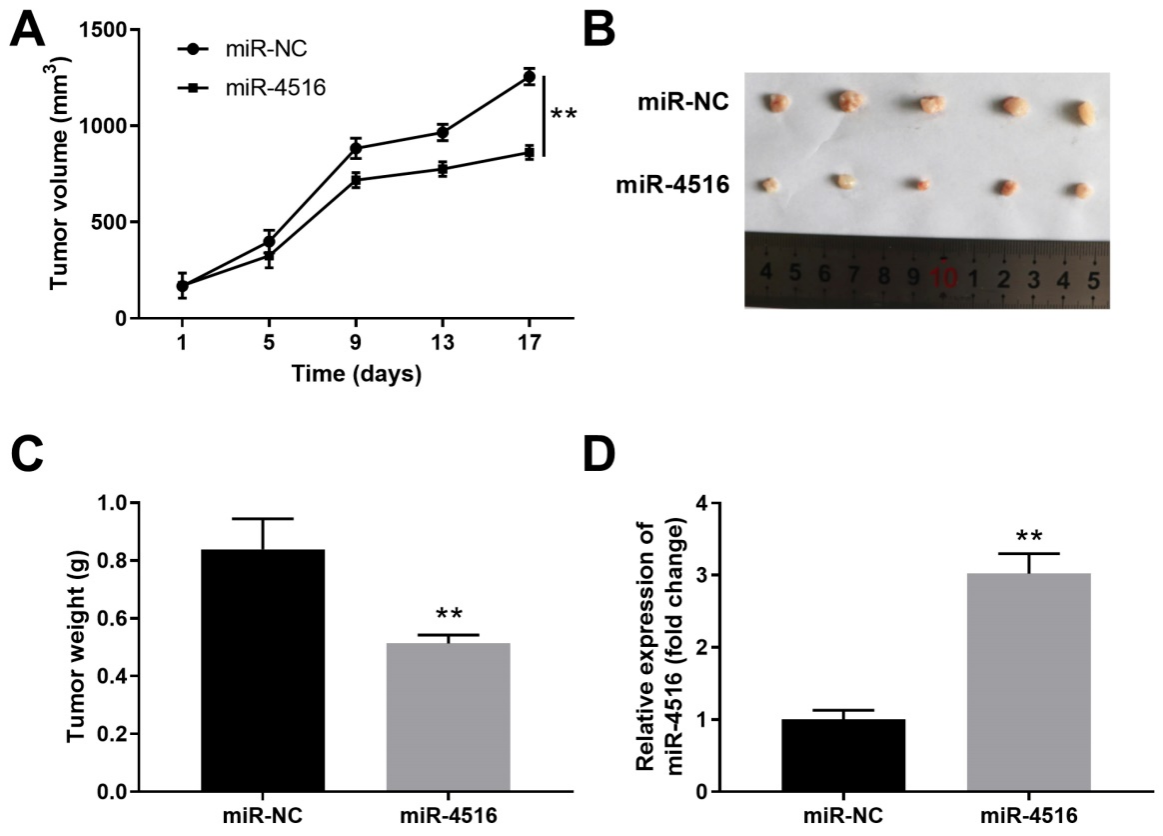
** MiR-4516 overexpression inhibits pancreatic xenograft tumor growth in vivo.** PANC-1 cells were stably transfected with miR-4516 or negative control miR-NC and implanted into the right flank of BALB/C nude mice. (A) Tumor growth was monitored and the tumor volume was assessed at indicated time points. (B, C) Xenograft tumor from miR-4516 overexpression group and control group was extracted from mice. (B) Tumor size and (C) Tumor weight were analyzed. (D) The expression of miR-4516 was analyzed in xenograft tumors by qPCR. **, *P* < 0.01.

To gain further insight of the function of miR-4516 in pancreatic cancer, we established a pancreatic cancer xenograft tumor model. PANC-1 cells stably transfected with miR-4516 mimics or miR-NC were transplanted into the nude mice. Overexpression of miR-4516 resulted in suppressed tumor growth (**Figure 3A**). The xenograft tumors from miR-4516 overexpression groups showed smaller tumor size and lower tumor weight than that from control group (**Figure 3B-3C**). Consistently, we confirmed that miR-4516 was highly expressed in xenograft tumors from miR-4516 overexpression group (**Figure 3D**).

### MiR-4516 negatively regulates OTX1 expression by binding to its 3'-UTR

**Figure 4:**
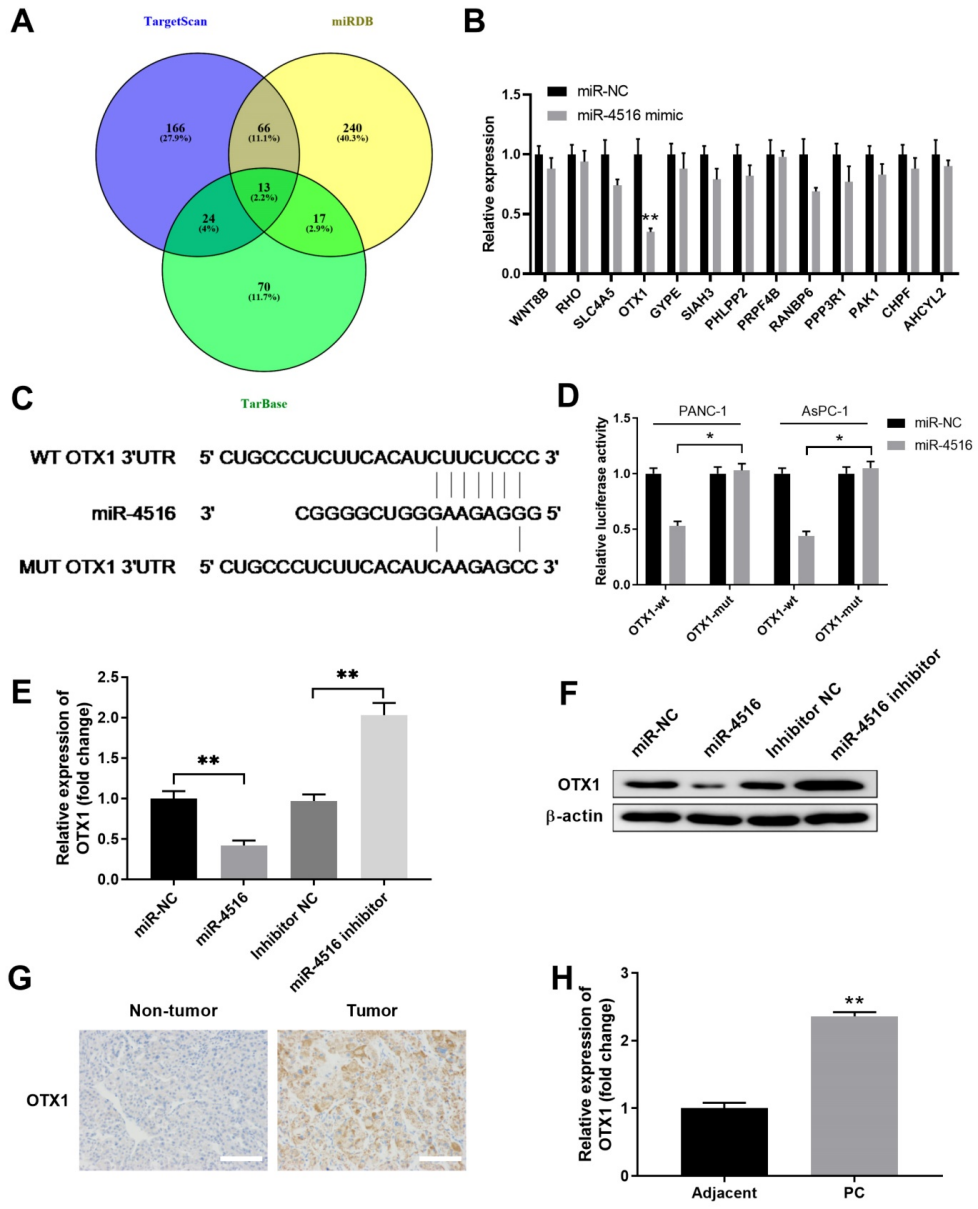
** MiR-4516 negatively regulates OTX1 expression by binding to its 3'-UTR.** (A) Bioinformatics analysis was performed to predict the potential targets of miR-4516 using online database TargetScan, miRDB, and TarBase. (B) PANC-1 cells were transfected with miR-4516 or miR-NC. Potential target gene expression levels were analyzed by qPCR 48 hours later. (C) The putative binding sequences between miR-4516 and WT 3'-UTR of OTX1 was shown. (D) PANC-1 or AsPC-1 cells were transfected with luciferase reporter vector containing WT or mutated 3'-UTR of OTX1, together with miR-4516 or miR-NC. The relative luciferase activity was analyzed 48 hours later. (E, F) AsPC-1 cells were transfected with miR-NC, miR-4516 mimics, miR-4516 inhibitor or inhibitor negative control. 48 hours later, the mRNA expression (E) and protein expression (F) of OTX1 were analyzed by qPCR and western blot. (G) The expression of OTX1 in pancreatic cancer tissues and adjacent non-tumor tissues was analyzed by immunohistochemical staining. (H) The relative expression of OTX1 in pancreatic cancer tissues and adjacent non-tumor tissues was analyzed by qPCR. *, *P* < 0.05; **, *P* < 0.01.

To understand the functional mechanism of miR-4516 in pancreatic cancer, we performed bioinformatics analysis using different online database to predict the potential targets of miR-4516. As shown in **Figure 4A**, combined analysis from TargetScan, miRDB and TarBase indicated 13 shared common targets. PANC-1 cells were transfected with miR-4516 mimics or miR-NC to verify the expression levels of these 13 targets. Overexpression of miR-4516 significantly inhibited the expression of OTX1 (**Figure 4B**). Further analysis revealed that miR-4516 had the complementary binding sequences targeting 3'-UTR of OTX1 (**Figure 4C**). Luciferase reporter vector containing wild-type (WT) or mutated 3'-UTR of OTX1 was constructed and transfected into PANC-1 or AsPC-1 cells together with miR-4516 mimics or miR-NC.

As expected, overexpression of miR-4516 specifically inhibited the luciferase activity in cells transfected with reporter vector containing WT 3'-UTR of OTX1, but not with reporter vector containing mutated 3'-UTR of OTX1 (**Figure 4D**). In addition, overexpression of miR-4516 inhibited OTX1 expression while inhibition of miR-4516 enhanced OTX1 expression in AsPC-1 cells at both mRNA and protein levels (**Figure 4E-4F**). Moreover, we found that OTX1 was highly expressed in pancreatic tumor tissues, as demonstrated by immunohistochemical staining (**Figure 4G**). Pancreatic tumor tissues also had notably lower levels of OTX1 mRNA compared with that in adjacent non-tumor tissues (**Figure 4H**). These findings indicated that miR-4516 might negatively regulate OTX1 by binding to its 3'-UTR.

### OTX1 is highly expressed in pancreatic cancer tissues and knockdown of OTX1 suppresses pancreatic cell migration and invasion

**Figure 5:**
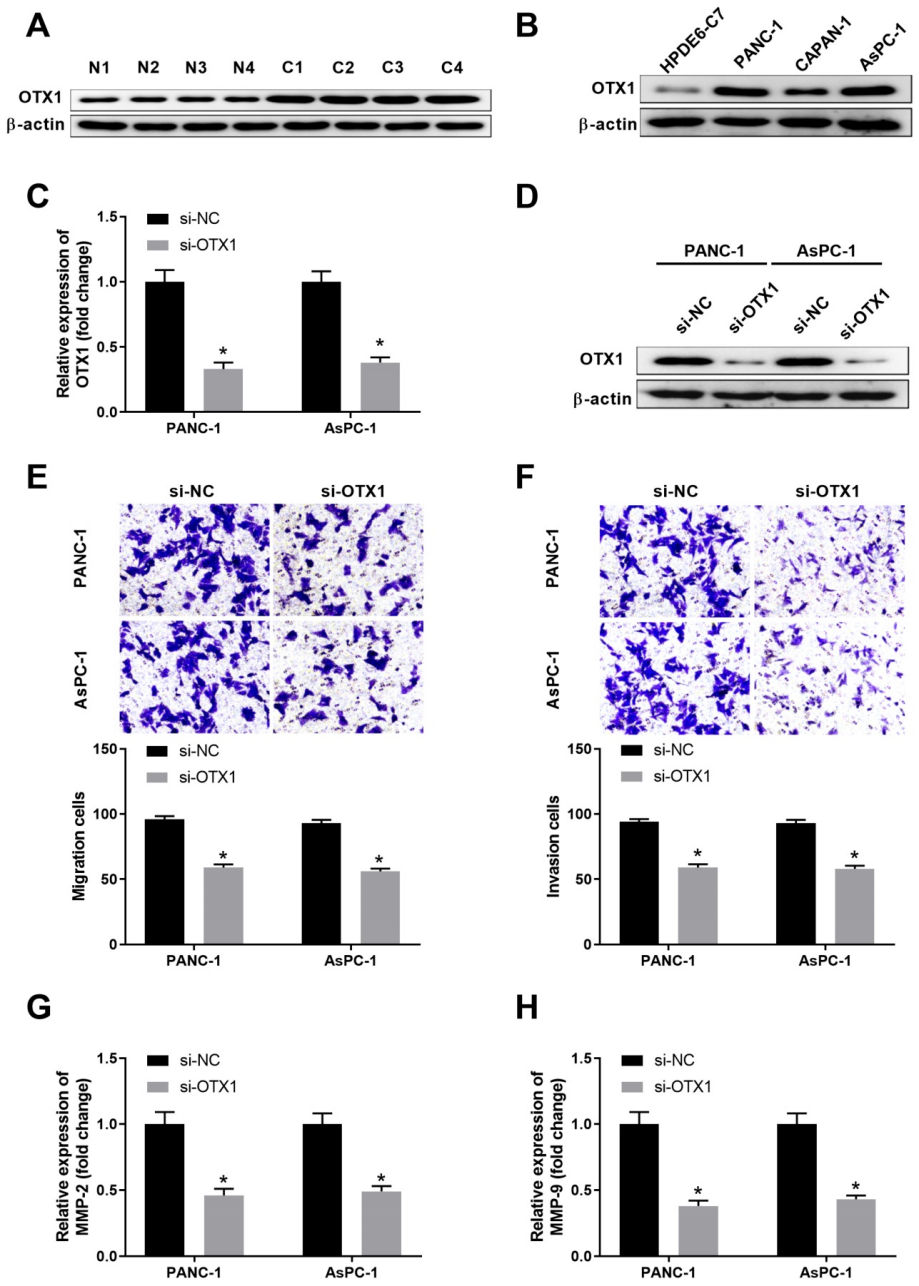
** OTX1 is highly expressed in pancreatic cancer tissues and knockdown of OTX1 suppresses pancreatic cell migration and invasion.** (A) The protein expression of OTX1 in pancreatic cancer tissues (C) or adjacent non-tumor tissues (N) was analyzed by western blot. (B) The protein expression of OTX1 in pancreatic cancer cell lines (PANC-1, CAPAN-1, and AsPC-1) and control cell line HPDE6-C7 was analyzed by western blot. (C, D) PANC-1 or AsPC-1 cells were transfected with si-NC or si-OTX1. The relative expression of OTX1 mRNA or the protein expression of OTX1 was analyzed by qPCR and western blot 48 hours later. (E, F) Transwell assay was performed to evaluate the migration and invasion capability of PANC-1 or AsPC-1 cells transfected with si-NC or si-OTX1. (G, H) PANC-1 or AsPC-1 cells were transfected with si-NC or si-OTX1. The relative expression of MMP2 and MMP9 was analyzed by qPCR 48 hours later. *, *P* < 0.05

We further explored the expression pattern and function of OTX1 in pancreatic cancer. As shown in **Figure 5A**, OTX1 was highly expressed in pancreatic cancer tissues compared with that in non-tumor tissues. Consistently, pancreatic cancer cell lines had significantly higher level of OTX1 than that in control cell line HPDE6-C7 (**Figure 5B**). RNA interfence was conducted and siRNA-targeting OTX1 notably repressed the mRNA and protein levels of OTX1 in PANC-1 and AsPC-1 cells (**Figure 5C-5D**). Transwell assay demonstrated that knockdown of OTX1 dampened cell migration and invasion in PANC-1 and AsPC-1 cells (**Figure 5E-5F**). Furthermore, OTX1 knockdown markedly inhibited the expression of MMP2 and MMP9 in PANC-1 and AsPC-1 cells, indicating that OTX1 played a critical role in pancreatic cell migration and invasion (**Figure 5G-5H**).

### MiR-4516 inhibits pancreatic cell growth, migration and invasion and promotes cell apoptosis via targeting OTX1

**Figure 6:**
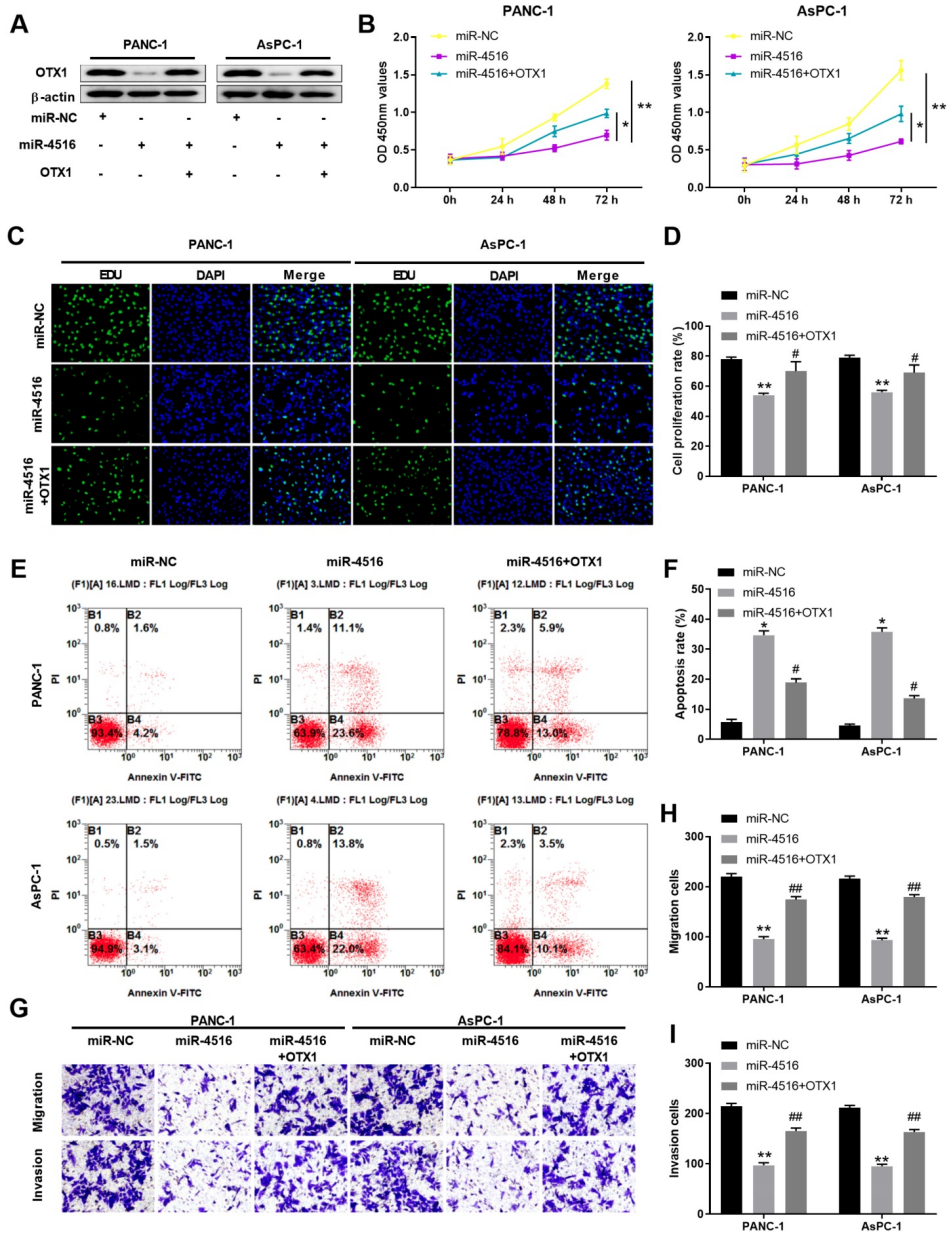
** MiR-4516 inhibits pancreatic cell growth, migration and invasion and promotes cell apoptosis via targeting OTX1.** PANC-1 or AsPC-1 cells were transfected with miR-NC, miR-4516 mimics, or miR-4516 mimics + OTX1 overexpression vector. (A) The protein expression of OTX1 in PANC-1 or AsPC-1 cells was analyzed by western blot. (B) Cell viability was analyzed by CCK-8 assay at indicated time points. (C, D) DNA synthesis was analyzed by EdU/DAPI double staining. (E, F) Cell apoptosis was analyzed by Annexin V/PI double staining. (G-I) Cell migration and invasion was analyzed by transwell assay. *, *P* < 0.05; **, *P* < 0.01.

To further verify the regulatory axis of miR-4516/OTX1, we transfected PANC-1 or AsPC-1 cells with miR-NC, miR-4516 mimics, or miR-4516 mimics + OTX1 overexpression vector. As shown in **Figure 6A**, miR-4516 mimics inhibited the expression of OTX1 while overexpression of OTX1 rescued the OTX1 expression in pancreatic cell lines. Overexpression miR-4516 repressed cell proliferation and DNA synthesis (**Figure 6B-6D**). However, overexpression of OTX1 partially reversed the inhibitory effect of miR-4516 (**Figure 6B-6D**). In addition, miR-4516 mimics promoted cell apoptosis while overexpression of OTX1 antagonized the promotion effect of miR-4516 mimics (**Figure 6E-6F**). Furthermore, transwell assay demonstrated that overexpression of OTX1 abrogated the inhibitory effect of miR-4516 mimics in pancreatic cell migration and invasion (**Figure 6G-6I**). Taken together, miR-4516 regulated pancreatic cell growth, migration, invasion, and apoptosis via targeting OTX1.

## Discussion

Mounting evidence has demonstrated that miRNAs play critical roles in tumorigenesis, function as tumor suppressors or oncogenes 8. MiR-4516 is a miRNA first identified to downregulate STAT3/CDK6/UBE2N signaling axis in PUVA induced apoptosis in keratinocytes 23. In our study, we found that miR-4516 was down-regulated in pancreatic cancer tissues and cell lines, indicating miR-4516 might be a tumor suppressor. We demonstrated that overexpression of miR-4516 inhibited pancreatic cancer cell proliferation, migration and invasion, while promoted cell apoptosis via negatively regulating OTX1. Furthermore, overexpression of miR-4516 suppressed tumor growth in vivo in pancreatic xenograft tumor model. These findings imply that miR-4516/OTX1 might be a useful therapeutic target in pancreatic cancer.

MiR-4516 was found highly expressed in dust-induced pulmonary fibrosis, which could be used as a potential biomarker for early diagnosis in patients with pneumoconiosis 24. In hepatocellular carcinoma, lncRNA LSINCT5 acted as a competing endogenous RNA and sponged miR-4516 in regulated HMGA2 expression 25. Interestingly, while our findings demonstrated that miR-4516 functioned as a tumor suppressor and inhibited pancreatic cancer development, miR-4516 is highly expressed in glioma patient samples and cell lines. Overexpression of miR-4516 promoted proliferation and invasion of glioblastoma cell via targeting PTPN14 15. The discrepancy of miR-4516 function in different tumors might due to the complicated tumor environment and regulatory network. A recent study revealed that miR-4516 overexpression suppressed the proliferation of breast cancer cells, especially triple negative breast cancer cells, indicating that miR-4516 might be used as an anti-cancer drug in breast cancer 26. In this study, both in vitro and in vivo experiments suggest that miR-4516 suppresses pancreatic cancer development and progression.

Combined bioinformatics analysis predicts OTX1 is the downstream target of miR-4516 in pancreatic cancer. Previous studies have shown that OTX1 was frequently upregulated in multiple cancers, including breast cancer, gastric cancer and colorectal cancer 18, 20, 27. Knockdown of OTX1 induced cell apoptosis, and attenuated cell migration and invasion of gastric cancer cells 20. In colorectal cancer, lncRNA FEZF1-AS1 regulated OTX1 expression by sponging miR-30a-5p 27. OTX1 was also reported to be negatively regulated by miR-3196 and lncRNA ADPGK-AS1 in breast cancer 27. We demonstrated that miR-4516 specifically down-regulated OTX1 expression in pancreatic cell lines. Lucifease reporter assay confirmed the posttranscriptional regulation of OTX1 by miR-4516. Moreover, miR-4516 exerted its tumor suppressor function via regulating OTX1 and overexpression OTX1 abrogated the effect of miR-4516 overexpression. However, whether OTX1 was also regulated by other miRNAs or lncRNAs remains to be further studied. Studies have demonstrated that OTX1 was involved in regulating ERK/MAPK signaling in hepatocellular carcinoma and p53 signaling in breast cancer 18, 28. The function mechanism of OTX1 in pancreatic cancer is not clear.

In conclusion, our findings suggest that miR-4516 acts as a tumor suppressor in pancreatic cancer. Overexpression of miR-4516 inhibits pancreatic cancer cell proliferation, migration, and invasion and promotes cell apoptosis via negatively regulating OTX1. Therefore, miR-4516/OTX1 might serve as a novel therapeutic target for miRNA-based therapy in pancreatic cancer.
